# Option-based guarantees to accelerate urgent, high-risk vaccines: a new market-shaping approach

**DOI:** 10.12688/f1000research.26482.2

**Published:** 2021-01-11

**Authors:** David Manheim, Derek Foster

**Affiliations:** 1 Health and Risk Communication Research Center, University of Haifa, Haifa, 3498838, Israel; 2 Rethink Priorities, Redwood City, CA, 94063, USA

**Keywords:** Vaccines, Advance market commitments, Health economics, Financing mechanisms, Public Policy

## Abstract

Accelerating the availability of COVID-19 vaccines is critical to preventing further waves and mitigating the impact on society. However, preparations for large-scale manufacturing, such as building production facilities, are typically delayed until a vaccine is proven safe and effective. This makes sense from a commercial perspective, but incurs great costs in terms of lives lost and damage to the economy. Several policy options are available to reduce this delay, all of which involve incentives or subsidies to invest in production facilities. We review existing approaches, then propose a novel alternative using “option-based guarantees” in which the government commits to paying a proportion of the manufacturer’s preparation costs should the product turn out not to be viable. Counterintuitively, this “payment for failure” is appropriate because in the case of success, a company makes a profit from the product itself, and does not need additional money from the government. While other approaches have critical roles, we argue that option-based guarantees are the most promising approach to ensuring a rapid vaccine for COVID-19. Compared to the alternative approaches, they reduce both costs to the government and risk to the companies, while maintaining an incentive to produce a high-quality product quickly and at scale.

## Introduction

The urgent need for a COVID-19 vaccine was widely recognized, with governments setting ambitious targets for timelines, testing, and approval. Even now that early candidates have been approved, more doses and likely additional vaccines would be useful in accelerating supply. A recent op-ed by Nobel Prize winner Michael Kremer and colleagues ^1^ noted that “we need multiple shots on goal” because a 90% chance of finding a successful vaccine requires funding 15–20 candidates. While the top candidates were already pushing forward with manufacturing well in advance of clarity about efficacy, longer-term safety cannot yet be evaluated, costs are high, and some are likely to be difficult to deploy to developing countries due to refrigeration requirements.

In addition, there are economic issues that proved to be critical barriers for vaccine development. It is not coincidental that despite hundreds of candidates which were quickly announced, the largest companies were by far the fastest to conduct trials, and to begin manufacturing. Companies with a smaller chance of being first to market have little incentive to invest in very expensive production facilities before their product achieves regulatory approval ^2^. Even now that top candidates are nearly certain to be deployed, at least for emergency use, scaling up production of alternative vaccine candidates is likely to add months to the overall timeline, costing thousands of lives and many hundreds of billions of dollars of lost GDP – far more than the cost of preparing to manufacture products that do not end up reaching the market.

### Many candidates

Perhaps more concerning, the first successful vaccine is not necessarily the best option.

Vaccine efficacy can vary widely. While the world was lucky that mRNA vaccines and other early vaccine candidates proved very effective, it was by no means obvious that one of the front-runner candidates would be approved. The FDA initially announced that it will approve vaccines which are only 50% effective, far short of the level needed for herd immunity, much less near-full protection ^3^. If early vaccines had been in fact only partially effective, or those vaccines are found to have longer term safety issues that present major drawbacks despite approval, investment in alternatives may still become a priority. And even if none of these problems occur, supplies of the initial groups of vaccine candidates will be in short supply for months, because the delay in investment has led to delays in scaling up. For the same reason, there will be delays in the production of any additional candidates which are found safe and effective.

A more diverse portfolio of options reduces the risk that a small group of vaccines are all insufficient, or that none are safe or effective enough to be approved. Even if this risk were low, the costs of failure would be high enough to warrant additional efforts. A critical task for governments is therefore to reduce delays due to risk-avoidance by firms so that multiple vaccines are developed and manufactured, thereby increasing the probability of finding a viable candidate and accelerating its availability. Before introducing solutions, however, it is worth reviewing part of the current landscape on which these solutions will operate.

### Vaccine development funding and market failures

There are a variety of mechanisms by which biomedical research, including vaccines, can be funded. This starts with the funding of basic research by governments, universities, and foundations, and continues through to mechanisms we discuss below for supplementing extant measures. In between, there is translational research, corporate research and development, and production costs. The funding for these stages comes from capital markets or private investments, or from nonprofits, nongovernmental organizations, and governments.

Despite these funding sources, there seem to be market failures, where socially beneficial products like vaccines are not produced because the firm-level incentives are insufficient to overcome the firm-level costs and risks.

It is useful to distinguish between two market failures that are implicated here. The first is a buyer/seller mismatch, where a market would exist for a product, but producers are unaware, or are concerned that the market will not buy sufficient quantity to justify investment. The second is a risk tolerance mismatch, where the socially optimal level of risk-taking is higher than the level for individual firms, so firms will not take risks in developing something that may not be purchased. This mismatch is particularly acute for smaller firms that cannot afford to absorb the loss from failures, but it also applies to larger firms, which may have high opportunity costs of capital.

## Potential solutions

For a pressing need like COVID-19 vaccines, a solution to the market failure provided by government or another organization must address multiple economic problems simultaneously. First, it needs to create incentives for companies and investors to take on high-risk projects, many of which have individually low probabilities of success. Second, it needs to create a motivation to scale up production capacity before the success of different approaches is known, to ensure timely availability. Finally, it should do these two things in ways that do not incentivise throwing money at projects that are too unlikely to succeed.

These different issues apply differently to different firm types, of course. Smaller firms have less capital, and less ability to raise capital, as well as a lower ability to diversify against the risk of long-shot projects. They are also far less able to invest in manufacturing. Larger firms, on the other hand, are far better at regulatory capture and finding ways to benefit from programs that could allow wasteful spending.

Several classes of solutions have historically been used to foster innovation: prizes, government programs, guaranteed demand, and for vaccines specifically, advance market commitments. While all are limited in their ability to compress timelines, each has significant advantages in addressing at least one of the market failures, or is useful for some firm types. A new approach we propose, option-based guarantees, seems particularly well suited to mitigating the risk tolerance mismatch for smaller and newer firms, some of which are pursuing novel ideas. This could have been critical to ensure a diversity of COVID-19 vaccine candidates, and could still foster innovation in vaccines more broadly.

### Prizes

Governments and private philanthropists can offer financial rewards for breakthroughs or solutions to a scientific problem. In the current scenario, a prize could be offered to companies that have a vaccine approved. Alternatively, companies could compete to offer the best idea for rapidly scaling up vaccines, and a contract to produce them could be part of the prize. By only paying out for successful solutions – or perhaps not any, if certain criteria are unmet – prizes are a fairly inexpensive option that incentivises innovation. This approach has a long history ^4^, and has been used by governments recently in the US ^5^ and abroad ^6^.

These cases illustrate that prizes are useful for spurring new areas of research, but primarily attract large firms or well-funded new entrants. This is partly because they require investors to supply and risk capital, while paying nothing to firms that “lose,” thereby failing to address (or worsening) risk tolerance mismatches. This is particularly difficult for smaller firms. The hope is that many firms would participate due to the increased gain in case of success, but there will not be adequate financial incentive for them to do so unless the probability of success is high, the investment is nearly viable without a prize, or the size of the prize makes the investment an expected gain despite the risk. These conditions seem unlikely to be met in the current circumstances, where it is likely that only a few of the many vaccines will ever be made widely available.

Another disadvantage relates to timing. Prizes are often appropriate for early-stage investments in projects that have little short-term chance of profitability, but which have a clear path to success. In the current situation, where speed is critical, the decision would ideally be immediate and certain, rather than contingent and post-success. This means that prizes do not address the risk mismatch issue. Not only this, but unlike the alternatives outlined below, because they are contingent upon success, prizes cannot be used or borrowed against to fund the project.

These drawbacks mean that, in the present case, prizes are unlikely to lead to a diversity of high-risk approaches.

### Government programs and public-private partnerships

Another alternative is the “Apollo program” or “Manhattan Project” approach, where the government directly invests massively in projects. Government agencies often enter into agreements, as the US Biomedical Advanced Research and Development Authority and others have done for COVID-19, to develop treatments, vaccines, and diagnostic tools. But this approach requires government expertise, and requires selecting one or a few projects to focus on. As a consequence, projects that are not funded, which are the majority of projects, languish unless and until the primary projects fail. It also favours well-connected and larger firms.

A common variant of direct government investment is public-private partnerships (PPPs). These share the risk between government and private companies, which allows the government to leverage private companies’ expertise. PPPs can be an effective means of achieving social objectives, but such deals generally take a long time to negotiate and implement, are complex in ways that can make regulatory capture a larger problem, and are usually best deployed when a single approach is needed.

Thus, a government-led approach is promising for relatively predictable projects, such as building test-and-trace infrastructure. In contrast, a key goal of higher-risk investments in vaccine candidates is to build a diverse portfolio of investments with an overall high probability of ensuring needs are met more quickly than markets allow on their own. Because multiple approaches are needed and the negotiation and development process is limited by government capacity, neither direct government funding nor PPPs are likely to be the best option for creating a large and diverse portfolio.

### Purchase orders and advance market commitments

Another approach is to pre-order vaccines. This has been done successfully by governments in the past, and in this case it could provide capital well before efficacy or safety is established. This would legally guarantee that producers have a market and that the company will supply the product, thereby reducing risk to both parties.

Kremer, Levin, and Snyder (2020) ^7^ present a version of this called advance market commitments (AMCs), which are purchase orders contingent on successful development. These have been used successfully for “technologically close products,” such as a pneumococcal conjugate vaccine. They are a particularly valuable tool when few of those who would benefit from a vaccine are able to afford it, in which case development is only economically feasible with an outside funder, though this does not apply to COVID-19.

However, this approach has several drawbacks, which the authors identify. First, both purchase orders and AMCs require choosing which approaches to fund or waiting until funding is not necessary to jumpstart production early. Governments’ track record of “picking winners” is less than stellar, and any such decisions would inevitably be highly politicized. Second, the government or other funder would need to negotiate prices and contractual details before companies would be able to start. Not only might this be a lengthy process, but vaccine manufacturers have a significant advantage in such negotiations, and there is potentially significant room for regulatory capture or windfall profits on the part of companies.

In addition to these problems, for COVID-19 this approach requires the government or other sponsor to commit to purchases of many still-unproven products. For this reason, it would need to contract with many different companies – the more the better, to improve the chances of a viable product – but committing to purchasing many vaccines when only a few will be needed would be very wasteful. In addition, purchase orders would potentially involve commitments to purchasing products not shown to be safe, which may be illegal for government agencies. It also gives far less incentive for producers to improve quality, speed, or cost-effectiveness through innovation. In the present crisis, these shortcomings are especially pressing.

A variant on AMC proposed by Athey *et al.* ^8^ to address COVID-19 would combine the direct investments (“push”) of PPPs with the typical AMC mechanism of a precommitment to purchase (“pull”) the first resulting product to come to market. This push-pull approach improves on both direct investment and PPPs; but because the government funding for purchasing may be exhausted before it reaches market, it has drawbacks similar to prize competitions in that it leaves companies with the bulk of the risk from overproduction if they are not first to market. This means that AMCs are an option regardless of which “push” option is selected.

## Option-based guarantees

In March and April of 2020, we suggested ^9^ a new approach for governments to “push” vaccine production, which is to enter into agreements with companies using put options. A put option (as in “put up for sale”) gives the holder the right, but not the obligation, to sell an asset, by (or on) a specified date, to the provider of the put. In this case, the put option would give companies the right to sell a portion of their investment in vaccine production to the government, i.e. at a guaranteed loss. Because there is no obligation to exercise the put, companies could sell viable products as usual, and would only use the option if their product turns out to be non-viable. If structured well, options can also align incentives in several other ways.

To understand how this would work, we start with an example, then note possible variants on the idea. Following this, we discuss the advantages of the proposal, and the implementation and political challenges it may face.

### Illustrative example

Suppose, optimistically, that a manufacturer thinks it will be able to produce 100 million doses of a vaccine within six months, but is delaying investment in production facilities because the vaccine’s Phase 3 trial results will not be available for a year. Once the result is known, it will begin to invest in the production, and if there are no unforeseen obstacles, have the vaccine available six months later.

Under the proposed scheme, the company can approach the government with a budget and a timeline, and the government can agree to provide a put option that allows the company to recoup, say, 90% of its eventual costs, capped at the company's initial project cost estimate, in exchange for the facility and equipment. If the vaccine is viable, the company would not exercise the option, the government would pay nothing, and the company would be able to sell the vaccine normally. If found non-viable, however, the company would have an incentive to stop production and exercise its option as soon as possible. When the option is exercised, a financial audit of costs would take place, and the government would accept delivery of any items purchased, built, and/or produced in exchange for 90% of costs. Delivery upon contract termination is both a potential avenue for the government to recoup costs, and a means to ensure companies do not gain windfall profits from declaring a program a failure, then selling assets.

### Variants

A number of variants of this approach are possible, and three are worth highlighting: declining payouts, priced contracts, and conditions on sales. The first two modify the incentives, while the final variant addresses additional concerns about the availability and price of the vaccine.

First, the payout for the put options could be declining over time, so that the payment is, say, 95% at the outset, and declines by a specified percentage, say 1%, each month. This will incentivize companies to exit as soon as possible if they think the project will fail.

Second, instead of providing options to companies for free, the government could charge for the contracts. This would further dissuade unqualified or undercapitalized companies from taking huge immediate risks with small probabilities of success. Prices for such contracts would still need to be a small fraction of the actuarially fair price, otherwise the scheme does not actually provide the needed incentives.

The last of the variants, conditions on sales, is somewhat different, since it is largely unrelated to the options themselves. Put options do not ensure the final vaccine is available at a reasonable price, but nor do they preclude other policy solutions. Because recipients of these options benefit from the program’s guarantee, in exchange for participation the government may claim priority for purchases, cap the profit margin on sales to the government, or cap the price paid by the government for a product. This is reasonable, but care should be taken not to either significantly reduce the incentive to invest, or greatly slow down the process of agreeing to deals. Note also that governments that took on risk to ensure investment in a product might also want to prioritize domestic purchases, rather than allowing them to be sold internationally. While posing additional challenges for international cooperation, this does not differ from other solutions, and can be addressed in similar ways, such as through international coalitions and agreements. Finally, we note that it may be less than ideal to pursue multiple goals with a single policy. If price controls or similar constraints are desired, they do not need to be tied to funding mechanisms.

## Discussion

The use of put options to accelerate vaccine production is a somewhat novel idea. Though it creates incentives and shapes markets in ways similar to other policy tools, it is worth looking at the unique advantages and drawbacks of this approach.

### Advantages

First, commercial companies can continue to use traditional methods for financing and operating their businesses without unnecessary government supervision or contracting. New companies can also use these options to help them secure funding from private investors, making the program more equitable to newer firms without requiring direct government investments.

Second, it provides incentives for starting production earlier, but preserves normal market mechanisms to provide high-quality products. Because the market is competitive, and it will be unclear whether other firms will be earlier to market or have a safer product, perhaps with higher efficacy, having an earlier and/or better product on the market will increase sales, and therefore profit.

Third, this approach is guaranteed to have lower cost than direct investment to pay for high-risk products, while preserving market incentives. Increasing the possible cost savings, the government may also be able to resell some items. For example, a plant or equipment designed to manufacture an ineffective vaccine could be resold and adapted to produce a different one. There have been intermittent shortages of other vaccines, so excess capacity may not be entirely wasted.

Fourth, unlike advance payments or contracts, put options do not subsidize companies to undertake projects that they expect cannot succeed, but do allow them to take additional risks in order to accelerate production.

Lastly, it can be implemented more quickly than the alternatives. Private funding that relies in part on the known risk reduction from the government guarantee could replace direct payment by the government. Not only that, but because the calculation of the payment is deferred, the approach could potentially be implemented without extensive and slow negotiations – a very important consideration in the current circumstances where speed is critical.

### Implementation challenges

The most critical decision for option-based guarantees is the structure of the payments. There is a tradeoff between payment amounts and incentives for firms. The ideal percentage of costs to reimburse with such a program requires economic analysis, weighing the cost of such a program, which likely involves payment of all or all but one of the put options, against the public benefit of a more rapidly available vaccine.

However, these challenges are not unique to put options. Any incentive for production requires the government to choose projects to fund, and then pick a level of funding. By guaranteeing the payout will be less than the investment, providing incentives for early termination, and enabling cost-recovery through reselling assets, put options reduce the risk of corporate profiteering relative to direct investment and PPPs. In addition, as noted above, AMCs and prizes are compatible with put options as well as those two alternatives.

Perhaps a greater disadvantage is that this is a novel suggestion: similar programs have not, to our knowledge, been tried before. While the proposal has attempted to consider implementation, unforeseen challenges may arise.

### The political challenge

An option-based guarantee is a potential political liability. The program may appear wasteful because, perhaps counterintuitively, payments are only made for unused products and abandoned projects. This may make the program politically unpopular, especially if no successful vaccines are generated, or if the costs outweigh the value of the successes. In addition, put options do not address pricing or local supply, so they do not guarantee that any viable vaccines would become widely available. This potentially makes options far better for funding research or manufacturing capacity, rather than the products themselves.

At least four factors should mitigate the political risk. First, as explained above, the payment structure should help to minimise waste, giving less ammunition to opponents. Second, the program could (quite accurately) be presented as evidence of government action to combat the pandemic, which is likely to be popular in the current climate. Third, the government could of course claim credit for any successful products emerging from the program – products that would save or improve many of their constituents’ lives. Fourth, additional mechanisms or clauses in the contract, such as the variants described above, could ensure that the products are sold at a reasonable price.

## Conclusion

The optimal approach – or more likely, combination of approaches – to developing healthcare products will vary by disease, time period, and urgency. For this reason, we conclude with a discussion of a few contexts in which option-based guarantees seem most useful, as well as areas where the other reviewed approaches are likely to be superior.

In the case of vaccines for COVID-19, we think that option-based guarantees for constructing production facilities would have been, and still may be, the best alternative for candidates that are promising but whose viability, large-scale manufacturing methods, and/or quantity required are substantially uncertain. These types of guarantees are potentially useful in other areas as well, but the selection of funding mechanisms should be made on the basis of the needs and characteristics of each specific product type and need, both in combating COVID-19 and for future pandemics.

We also think that option-based approaches may be useful for funding very costly Phase 2 and 3 trials, perhaps in place of the current model of directly funding large firms. This is especially true for smaller firms that may otherwise delay or under-power vaccine trials to mitigate risks. In this case, they could be paid part of their costs if the product fails to gain approval.

Based on our review, PPP or direct purchase orders are much more appropriate than option-based guarantees for low-risk products. For example, purchasing a large number of a certain vaccine that is already near approval would be ideal if the safety, accuracy, cost, and quantity required are known, the company is trusted, and the paperwork can be done quickly. Antivirals or antibiotics that are already being produced and are likely to be useful may also fall into this category. This approach can also mitigate the risk that a company will be slow to respond to anticipated but uncertain demand.

Athey *et al.*’s advance market commitments are useful when products are higher risk, but are close enough to being ready that price and quantity negotiations can take place before the decision is made. In such a case, option-based guarantees are less helpful.

In other contexts, such as when innovative solutions with low capital costs but significant conceptual innovation are likely to be needed, prizes for successful innovation are another useful approach for creating incentives for investments. This would potentially be true for new types of point-of-care tests for active infection and new monitoring technologies. Both AMCs and prizes are also useful in combination with any of the proposed “push” funding approaches, including option-based guarantees.

Over the coming weeks and months, choosing the right funding mechanisms could save tens of thousands of lives. The sooner additional companies start these investments, the sooner their products can reduce economic damage, mitigate ongoing risks to the safety of vulnerable communities, and staunch the very high human costs of the ongoing COVID-19 pandemic.

## Data availability

No data is associated with this article.
