# Supplementary material for: Option-based guarantees to accelerate urgent, high-risk vaccines: a new market-shaping approach
Source: F1000Res. 2021 Jan 11;9:1154. Originally published 2020 Sep 18. [Version 2] doi: 10.12688/f1000research.26482.2 (PMC7808051; doi:10.12688/f1000research.26482.2)
Supplement: Peer review history

# Peer review: Reviewer response for version 2

**DOI:** 10.5256/f1000research.31444.r77016

**Published:** 2021-01-13

**Authors:** Rino Rappuoli, Simone Pecetta

**Affiliations:** 1 GlaxoSmithKline (GSK), Siena, Italy; 2 GlaxoSmithKline (GSK), Siena, Italy

Rino Rappuoli: I have no additional comments.

 Simone Pecetta: I also have no additional comments and I believe the manuscript is suitable for indexing.

Is the topic of the opinion article discussed accurately in the context of the current literature?

Yes

Are arguments sufficiently supported by evidence from the published literature?

Yes

Are all factual statements correct and adequately supported by citations?

Yes

Are the conclusions drawn balanced and justified on the basis of the presented arguments?

Yes

Reviewer Expertise:

NA

We confirm that we have read this submission and believe that we have an appropriate level of expertise to confirm that it is of an acceptable scientific standard.

# Peer review: Reviewer response for version 1

**DOI:** 10.5256/f1000research.29239.r74620

**Published:** 2020-11-26

**Authors:** Rino Rappuoli, Simone Pecetta

**Affiliations:** 1 GlaxoSmithKline (GSK), Siena, Italy; 2 GlaxoSmithKline (GSK), Siena, Italy

The paper proposes one alternative mechanism to increase the incentives to develop vaccines which do not have an attractive market. The authors call this mechanism “option-based guarantees.” Overall, proposing an additional model to develop vaccines when there is a market failure is fine, because we need as many options as possible. 

 Here are some comments:

1. This proposal is too late for COVID-19 vaccines, as they have been already developed.
2. The authors argue for an option-based approach to help manufacturers in investing in new vaccines by de-risking eventual failures with public money. This approach might be relevant to incentivize development efforts in areas of high public health and medical need.
3. Overall, the manuscript is well written and brings interesting insights on mechanisms that will likely be at the center of the public attention in the upcoming post-discovery phase of COVID-19 vaccines. However, its content seems to be completely detached from present day issues and it is very theoretical. It is not clear how the proposed solution integrates with - or differentiates from - the dozens of private and public initiatives that support COVID-19 vaccine development. Are there already option-based approaches being used for COVID-19 vaccines? If not, why? Are governments or public entities considering such approaches for some of the vaccines under development?

Minor: the authors should update the introduction session as - as of today - two vaccine candidates have already demonstrated high efficacy in interim ph3 analysis without the need of human challenge trials and with much stringent timelines than reported in this manuscript.

Is the topic of the opinion article discussed accurately in the context of the current literature?

Yes

Are arguments sufficiently supported by evidence from the published literature?

Yes

Are all factual statements correct and adequately supported by citations?

Yes

Are the conclusions drawn balanced and justified on the basis of the presented arguments?

Yes

Reviewer Expertise:

infectious diseases; vaccines

We confirm that we have read this submission and believe that we have an appropriate level of expertise to confirm that it is of an acceptable scientific standard, however we have significant reservations, as outlined above.

# Response

**Published:** 2020-12-14

**Authors:** David Manheim

**Affiliations:** University of Haifa, Israel

Thank you very much for the helpful notes and concerns. We are in the process of finalizing a revised version of the manuscript that addresses the concerns. We respond to the individual points below.

**1. This proposal is too late for COVID-19 vaccines, as they have been already developed.**

 While there are now approved vaccines, manufacturing for COVID-19 vaccines is not obviously a fulfilled need, and still-unapproved second-generation vaccines may still have an important role in fulfilling global demand. At the same time, we agree that at the current time, the proposal is far less relevant. At the time it was proposed and initially discussed with policymakers, this was not yet true. The current paper is the culmination of an initial idea from the beginning of April [1], a presentation of the idea in mid-April [2], discussions with policymakers, and an initial writeup [3] which was discussed seriously, albeit privately, as a policy approach. We have now cited the writeup in the revision of the paper. The peer reviewed publication process is unfortunately far slower than the policy process, and the current manuscript has been a victim of that delay.

 We will note that the developed vaccines are still being manufactured far more slowly than would be ideal. An investment of several billion dollars several months ago, earlier during the trials, would have significantly alleviated the current lack of vaccine production capacity. For that reason, it still seems very relevant to the policy discussion to point out that mechanisms to allow this were being proposed in time for them to have been used. 

 Moreover, it is clearly relevant as a possible approach for future crises. It would be a shame if promising ideas once again only gained traction in academic and policy circles when it was too late to avert catastrophic outcomes.

**2.** **The authors argue for an option-based approach to help manufacturers in investing in new vaccines by de-risking eventual failures with public money. This approach might be relevant to incentivize development efforts in areas of high public health and medical need.**

 We certainly agree that this approach is viable in some other contexts, and agree that the paper is useful for that context. As we discussed in the paper, the different approaches are relevant for different needs.

 We are skeptical, however, that the approach we suggest for COVID-19 is applicable widely outside of the urgent development of a new and promising technology to meet a widely recognized need. We can certainly envision scenarios where the mechanism would be viable, but because the primary advantage is de-risking capital investment, rather than funding development, it seems unlikely to spur great interest unless and until there is a new need for drastic action, or a niche use case can be found to pioneer the method on a smaller scale via a philanthropic investment.

**3. Overall, the manuscript is well written and brings interesting insights on mechanisms that will likely be at the center of the public attention in the upcoming post-discovery phase of COVID-19 vaccines. However, its content seems to be completely detached from present day issues and it is very theoretical. It is not clear how the proposed solution integrates with - or differentiates from - the dozens of private and public initiatives that support COVID-19 vaccine development. Are there already option-based approaches being used for COVID-19 vaccines? If not, why? Are governments or public entities considering such approaches for some of the vaccines under development?**

 We appreciate the feedback. The paper presents a mechanism, and requires policy-decision making and negotiation if it were used in the future to integrate with other mechanisms. The mechanisms which were used seem to have spurred sufficient investment, though it is unclear how much of the credit for this should go to the financing mechanisms.

 While we will not discuss the history and context extensively in the paper, we will make a few observations here in the reply. As the paper discusses, the proposal is novel, and while we think there was promise, we are unaware of any earlier suggestions or current plans for using the novel mechanism. This seems to be due to a combination of factors. First, given the prominent role that the US played in financing vaccines, and the political turmoil of the Trump administration, there was reticence on the part of policymakers to try anything which might draw attention to the potential risks. Second, once funding was made available widely for vaccine development, efficiency of the mechanism was relegated to a minor role. 

 Given this, we note that despite thinking our proposed mechanism is promising for any future pandemics, over-supply of funding was potentially a better choice, and is certainly simpler. Almost any plausible level of overspending on the vaccines to spur faster development and production would have been worthwhile, post-hoc. At the same time, investment in production of vaccines was not as timely or as large as could have been achieved with this model. Soon after this model was first proposed, in April, the Gates Foundation announced it would fund production of the 7 leading vaccine candidates, and it did so by funding GAVI to enter into advance market commitments.

The EU similarly used AMCs, which were paired with funding for production. Unfortunately, as we discussed in the manuscript, the funds are only useful for providing capital, in these cases, to already-large global firms with existing access to global capital markets. Because the purchases are contingent on approval, they do not decrease risk, and so production has been slow. This makes sense for firms; if the purchase agreement is early, the company bears the risk of it not being approved - as seems to have occurred recently in Australia with the UQ/CSL vaccine, where the advance purchase was cancelled.

 Moreover, because AMCs are only used after it seems clear a vaccine has a high chance of success, these were not entered into until after June. The stated rationale was that “this Strategy is therefore similar to an insurance policy, by transferring some of the risks from industry to public authorities in return for assuring Member States of equitable and affordable access to a vaccine.” The proposal put forward here is a more straightforward way to accomplish the first half. The ethics of ensuring supply to some countries at the expense of others aside, as we noted, this does not preclude also using purchase commitments to guarantee supply. Not only this, but provision of funds was slow. Despite early promises, it took until August for the Gates Foundation to give $150m to increase production in India.

**Minor: the authors should update the introduction session as - as of today - two vaccine candidates have already demonstrated high efficacy in interim ph3 analysis without the need of human challenge trials and with much stringent timelines than reported in this manuscript.**

 This is now edited in the revision to reflect the timing of the publication.

 Citations:

 1. Manheim, D. (2020, April 2). A Simple Proposal for Jumpstarting Vaccine Production. In about 12 months, the world will need to start producing massive quantities... [Tweet]. Twitter. https://twitter.com/davidmanheim/status/1245810066843983872 

 2. Manheim, D. (2020, April 13). A Proposal to Accelerate Vaccine Production Now [Video]. YouTube. https://www.youtube.com/watch?v=mVjqGh_Dmv8

 3. Foster, D., & Manheim, D. (2020, April 27). Market-shaping approaches to accelerate COVID-19 response: A role for option-based guarantees? LessWrong Forum. https://www.lesswrong.com/posts/uXb4gcDP2fgBPcMHJ/market-shaping-approaches-to-accelerate-covid-19-response-a

# Peer review: Reviewer response for version 1

**DOI:** 10.5256/f1000research.29239.r73554

**Published:** 2020-11-09

**Authors:** Daniel L. Tortorice

**Affiliations:** 1 Department of Economics and Accounting, College of the Holy Cross, Worcester, MA, USA

This paper considers how governments can accelerate the availability of COVID-19 vaccines. Since investment in production facilities is a large risky investment, without government intervention vaccine manufacturers would be expected to delay this investment until after successful Phase 3 trials. Since this delay is socially costly, government intervention is justified. The authors propose a new financing mechanism to speed up investment. Specifically, the government will provide vaccine manufacturers with a put option, that if exercised, will require the government to purchase vaccine manufacturing equipment and facilities for a prespecified amount, for example 90% of the cost of developing the facilities. The paper then compares the proposed financing mechanism with existing financing mechanisms and discusses the relative advantages and disadvantages of the proposed mechanism.

 Accelerating the development of a COVID vaccine is one of the most pressing challenges of our times and I commend the authors for inventing and developing a new financial mechanism to aid in this acceleration. Their proposed mechanism is a clever compromise between the direct funding of manufacturing approach taken in the US and the advanced purchase commitment approach taken in Europe.

 I have two concerns about the proposed mechanism which I outline below and several minor points which might help the authors improve the article.

 The article does not discuss how manufacturers will be chosen to receive this option. Since the authors suggest using this mechanism to fund vaccine trials as well as manufacture, the number of companies that one might reasonably consider as applicants is quite large. The Milken Institute estimated there are 214 Covid-19 vaccines in development. Additionally, the option has no downside for the vaccine developer, so there is no reason they would not apply. The mechanism by which vaccine producers are chosen is essential. Because the option is worth more, the more likely you are to fail, offering the option will select producers with the least viable prospects. Additionally, given the difficulty of separating out project specific costs and costs the firms would have paid anyway, the option would attract firms with lower probabilities of success that see the option as away to increase general funding for their operations. The authors should make clear if and how they intend to limit the number of producers who receive the option.

 Another issue that could be discussed is the socially optimal number of vaccines. From a social perspective, we would like a large number of safe and effective vaccines. This is both because increased competition benefits consumers through lower prices and population subgroups may respond differently to various vaccines. This reason is why wealthy countries have invested in a diverse portfolio of vaccine and agreed to advanced purchase commitment. However, even if a vaccine is proven safe and effective, a vaccine manufacturer may exercise the put option if they view the vaccine market as crowded and not profitable enough even if additional vaccines would be socially beneficial.

 Some more minor points. [Note the first three points below are why I answered partly to the question “Are all factual statements correct and adequately supported by citations?”]

 A citation should be provided for the Michael Kremer op-ed article.

 The estimate of the monthly costs of the pandemic “tens or hundreds of billions of dollars of lost GDP,” seems too conservative. It is clearly in the hundred of billions of dollars per month even if you are only counting the US. For example, Cutler and Summers (2020) ^1^ puts it in excess of 800 billion per month.

 The claim that a vaccine with 50% efficacy is far short of the level of protection needed for herd immunity does not seem to be supported by the literature. See for example, Gomes *et al*. (2020) ^2^ which estimate 60-70% immunity as sufficient for heard immunity.

 I found the paragraph on page 4 about the timing of prizes lacking precise reasoning. For example, while they cannot be borrowed against, they can help vaccine developers find investors by increasing their profit if they produce a successful candidate. Additionally, the option the authors propose is both contingent and in the future, so this line of thought doesn’t really make a distinction between prizes and the option.

 It is incorrect to say AMCs require governments to pick winners. The typical AMC requires the government purchase the vaccine only when it is approved as safe and effective by a regulatory body like the FDA or WHO. As such it tends to select firms who believe they have a high likelihood of success.

 On page 5 there is a typographical error. “Put options do not ensure the final vaccine is available at a reasonable price, but nor do they preclude other policy solutions.” The word but is not needed.

Is the topic of the opinion article discussed accurately in the context of the current literature?

Yes

Are arguments sufficiently supported by evidence from the published literature?

Yes

Are all factual statements correct and adequately supported by citations?

Partly

Are the conclusions drawn balanced and justified on the basis of the presented arguments?

Yes

Reviewer Expertise:

Macroeconomics, Finance, Value of Vaccination

I confirm that I have read this submission and believe that I have an appropriate level of expertise to confirm that it is of an acceptable scientific standard.

## References

[1] : The COVID-19 Pandemic and the $16 Trillion Virus. *JAMA*.2020;324(15) : 10.1001/jama.2020.19759 10.1001/jama.2020.19759 33044484PMC7604733

[2] : Individual variation in susceptibility or exposure to SARS-CoV-2 lowers the herd immunity threshold. *medRxiv*.2020; 10.1101/2020.04.27.20081893 10.1101/2020.04.27.20081893 PMC885566135189135

# Response

**Published:** 2020-12-14

**Authors:** David Manheim

**Affiliations:** University of Haifa, Israel

Thank you for your review and comments. We have responded to the points raised below, and are in the process of posting a revision which clarifies many of these points in the text. We have responded to the two major points, then responded to each of the minor points below.

 First, the review notes: " *The article does not discuss how manufacturers will be chosen to receive this option. Since the authors suggest using this mechanism to fund vaccine trials as well as manufacture, the number of companies that one might reasonably consider as applicants is quite large. The Milken Institute estimated there are 214 Covid-19 vaccines in development. Additionally, the option has no downside for the vaccine developer, so there is no reason they would not apply. The mechanism by which vaccine producers are chosen is essential. Because the option is worth more, the more likely you are to fail, offering the option will select producers with the least viable prospects. Additionally, given the difficulty of separating out project specific costs and costs the firms would have paid anyway, the option would attract firms with lower probabilities of success that see the option as a[ ]way to increase general funding for their operations. The authors should make clear if and how they intend to limit the number of producers who receive the option."*

 We agree that there is some amount of discretion needed on the part of governments, but as we noted in the paper “these challenges are not unique to put options. Any incentive for production requires the government to choose projects to fund, and then pick a level of funding.” While the existence of 214 candidates seems daunting, it is immediately clear that some are more advanced along the track than others (and at this point, many are finished) so the government's choices can be far better informed. Lastly, as a policy analysis, we think the paper is more helpful explicitly leaving this choice to policymakers and the political process, where it will inevitably happen.

 However, the mechanism is not costless for manufacturers, since less than the full cost is paid. The option is more valuable to a less viable candidate, but because they have some portion of the funds at risk, despite a guaranteed partial repayment upon failure, they will be less interested in making an investment that is nearly certain to lose money, even if it is only a fraction of the investment.

 Additionally, the put option does not provide money upfront, and this means that most smaller manufacturers would need loans. Because of the very high cost of building manufacturing capacity, an option which repays “90% of costs,” as the paper suggests, would mean that failure is still likely to bankrupt smaller firms. For larger firms, we agree that there is a risk that they use the put option to reduce their cost of capital rather than spend more. For smaller firms, lenders will plausibly be willing to make such loans if there is an option in place, but it by no means suggests that lenders will give money to projects which are near-certain to fail – despite the ability to recover most of their investment due to the option, they are unlikely to be repaid in full in that case.

 For this reason, we think that the use of options will be limited to firms with a reasonable chance of success – and since only a finite number of vaccines are needed, the number of such firms which can succeed is, by nature of the problem, small.

 Second, the review notes that *"Another issue that could be discussed is the socially optimal number of vaccines. From a social perspective, we would like a large number of safe and effective vaccines. This is both because increased competition benefits consumers through lower prices and population subgroups may respond differently to various vaccines. This reason is why wealthy countries have invested in a diverse portfolio of vaccine and agreed to advanced purchase commitment. However, even if a vaccine is proven safe and effective, a vaccine manufacturer may exercise the put option if they view the vaccine market as crowded and not profitable enough even if additional vaccines would be socially beneficial."* 

 We agree that this could be a concern, but suggest two reasons it is unlikely. First, the manufacturer would be publicly claiming failure in a way that is nearly certain to create public backlash. Second, the public is by no means powerless in such a scenario – any philanthropist or government that wishes to see the vaccine produced has every right, and a significant incentive, to offer funds or contracts to urge a producer of, say, the 5th or 6th vaccine which is found effective and safe to continue despite their otherwise unprofitable situation.

 Regarding the more minor points, we have responded below.

**A citation should be provided for the Michael Kremer op-ed article.**

 Thank you - this oversight is now corrected.

**The estimate of the monthly costs of the pandemic “tens or hundreds of billions of dollars of lost GDP,” seems too conservative. It is clearly in the hundred of billions of dollars per month even if you are only counting the US. For example, Cutler and Summers (2020)1 puts it in excess of 800 billion per month.**

 Thank you for pointing this out - we have clarified. At the time the paper was initially written, the situation was less clear, and the estimate was clearly very conservative/optimistic.

**The claim that a vaccine with 50% efficacy is far short of the level of protection needed for herd immunity does not seem to be supported by the literature. See for example, Gomes et al. (2020) which estimate 60-70% immunity as sufficient for [herd] immunity.**

 The occasional discussion of successful immunization with lower numbers of vaccinated individuals rests on a number of very dicey assumptions. While the point is mostly irrelevant to the current paper, it is sufficient to note that, to be generous to the assumptions of those promoting the vaccination of superspreaders, it seems extremely optimistic to think that countries which cannot manage viable test and trace programs will nonetheless be able to identify the future superspreaders prospectively. This also ignores the fact that “superspreader” is a characteristic of the circumstances, and the constantly shifting set of contacts individuals have over time – not simply a property of an individual which can be found in advance. Beyond that, the assumption is that almost all of these (by assumption) foolhardy individuals – those most likely to contract and spread COVID-19 – would be willing to be vaccinated. 

 For a very recent preprint that lays out some of these issues, see Fox et al. [1]

**I found the paragraph on page 4 about the timing of prizes lacking precise reasoning. For example, while they cannot be borrowed against, they can help vaccine developers find investors by increasing their profit if they produce a successful candidate. Additionally, the option the authors propose is both contingent and in the future, so this line of thought doesn’t really make a distinction between prizes and the option.**

 The key difference between prizes and options is the combination of timing and contingency. As noted, prizes cannot be relied on or borrowed against, so even if they change the overall incentives to succeed, they won’t impact overall timelines as significantly. Options, because they are a financial guarantee which can be borrowed against, allow and encourage earlier and larger investments in production; the contingency ensures profitability, rather than enhancing it, as occurs in the case of prizes.

**It is incorrect to say AMCs require governments to pick winners. The typical AMC requires the government purchase the vaccine only when it is approved as safe and effective by a regulatory body like the FDA or WHO. As such it tends to select firms who believe they have a high likelihood of success.**

 We agree that not all AMCs require picking winners, and this is a tradeoff between early funding, and picking winners. We have edited to clarify that this is a tradeoff. By the time a typical AMC can be put in place, market funding is likely available, and it does not reduce risks. If the purchase agreement is early, the company bears the risk of it not being approved - as seems to have occurred recently in Australia with the UQ/CSL vaccine. 

**On page 5 there is a typographical error. “Put options do not ensure the final vaccine is available at a reasonable price, but nor do they preclude other policy solutions.” The word but is not needed.**

 Thank you. The British and American usage seem to differ slightly, and it can be used since this contrasts a positive and negative point, but we have amended it to omit the word.

 1. The COVID-19 herd immunity threshold is not low: A re-analysis of European data from spring of 2020 Spencer J. Fox, Pratyush Potu, Michael Lachmann, Ravi Srinivasan, Lauren Ancel Meyers

 medRxiv 2020.12.01.20242289; doi: https://doi.org/10.1101/2020.12.01.20242289
